# Aesthetic Experiences Across Cultures: Neural Correlates When Viewing Traditional Eastern or Western Landscape Paintings

**DOI:** 10.3389/fpsyg.2019.00798

**Published:** 2019-04-17

**Authors:** Taoxi Yang, Sarita Silveira, Arusu Formuli, Marco Paolini, Ernst Pöppel, Tilmann Sander, Yan Bao

**Affiliations:** ^1^Institute of Medical Psychology, Ludwig-Maximilians-University, Munich, Germany; ^2^Human Science Center, Ludwig-Maximilians-University, Munich, Germany; ^3^School of Psychological and Cognitive Sciences, Peking University, Beijing, China; ^4^Clinic and Policlinic for Radiology, Ludwig-Maximilians-University, Munich, Germany; ^5^Parmenides Center for Art and Science, Pullach, Germany; ^6^Physikalisch-Technische Bundesanstalt, Berlin, Germany; ^7^Beijing Key Laboratory of Behavior and Mental Health, Peking University, Beijing, China

**Keywords:** beauty, cultural identity, aesthetics, visual perception, fMRI, Western painting, Eastern paintings

## Abstract

Compared with traditional Western landscape paintings, Chinese traditional landscape paintings usually apply a reversed-geometric perspective and concentrate more on contextual information. Using functional magnetic resonance imaging (fMRI), we discovered an intracultural bias in the aesthetic appreciation of Western and Eastern traditional landscape paintings in European and Chinese participants. When viewing Western and Eastern landscape paintings in an fMRI scanner, participants showed stronger brain activation to artistic expressions from their own culture. Europeans showed greater activation in visual and sensory-motor brain areas, regions in the posterior cingulate cortex (PCC), and hippocampus when viewing Western compared to Eastern landscape paintings. Chinese participants exhibited greater neural activity in the medial and inferior occipital cortex and regions of the superior parietal lobule in response to Eastern compared to Western landscape paintings. On the behavioral level, the aesthetic judgments also differed between Western and Chinese participants when viewing landscape paintings from different cultures; Western participants showed for instance higher valence values when viewing Western landscapes, while Chinese participants did not show this effect when viewing Chinese landscapes. In general, our findings offer differentiated support for a cultural modulation at the behavioral level and in the neural architecture for high-level aesthetic appreciation.

## Introduction

Neuroscientific research on aesthetic processes in the visual system has offered evidence for a correspondence between certain properties of artworks and organizational principles in the brain (Zeki, 1999; Kawabata and Zeki, 2004; Chatterjee, 2011; Chatterjee and Vartanian, 2014; Bao et al., 2017a). Aesthetic experience partly relies on a visual analysis of an artwork based on distinct and specific visual attributes in color, line, texture, and form (Cinzia and Vittorio, 2009; Palmer et al., 2013). Indeed, some researchers (e.g., Latto, 1995; Bao et al., 2017b) have argued that artists implicitly tap into propensities of the human nervous system when generating aesthetic appeal. Piet Mondrian’s paintings are exemplary of visual art that excites orientation-selective neurons in the primary visual area. These neurons respond selectively to dots and straight lines, especially horizontal and vertical ones. Zeki (1999) principal idea is that different kinds of artworks excite different groups of neurons in the brain, leading to differential bottom-up processes.

Drawing upon complementarity as a basic principle of biological functions (Bao et al., 2017a), aesthetic experience relies both on higher-level cognitive processes and lower-level visual analyses to enable object identification, which is necessarily both bottom-up and top-down (Zhou et al., 2016). Some of the most studied modulators of the top-down system are individual expectations, prior knowledge, social context, and cultural background (Silveira et al., 2012, 2015b,c; Graupmann et al., 2013; Leder, 2013), which also applies to other cultural domains, like poetry (e.g., Zhao et al., 2018) or even religious beliefs (Silveira et al., 2015a). It has recently been suggested that aesthetic experience is partially top–down and varies between individuals according to their cultural experience (Redies, 2015). A match of internal and external information as represented in feedforward and feedback signals of the two complementary systems plays an important role in creating perceptual stability and even pleasure (von Holst and Mittelstaedt, 1950). Human cognitive architecture is presumably built to predict representations of the world and efficiently minimize prediction errors (Clark, 2013). Conceptually overlapping with this notion, art theory describes an aesthetic experience as an interplay between internal and external perspectives (Bao et al., 2017a), i.e., by dissolving the subject/object dichotomy, the observer experiences a sense of immersion (Heidegger, 1986).

In an attempt to resolve this bi-directionality or complementarity (Bao et al., 2017a), one approach is to define an artwork as a medium capturing both an artist’s understanding of a viewer’s experiences and a viewer’s understanding of an artist’s intentions (Jucker and Barrett, 2011; Tinio, 2013). This perspective considers aesthetic appreciation to be an interaction between an artwork’s objective properties and the viewer’s processing characteristics of those properties. This idea is consistent with suggestions that beauty is not “put in” the artwork by the artist as a distinct entity (Zaidel, 2015), but rather “an emergent property in the brain of the beholder,” where the beholder can include the artist, as well as the viewer (Redies, 2015). From a neuroimaging perspective, this phenomenon might be related to brain activation, which corresponds to the so-called mirror-neuron system and embodied simulations of emotions and actions (Freedberg and Gallese, 2007). It has repeatedly been found that positive aesthetic appreciation goes along with neural activation in parietal and sensory-motor brain regions (e.g., Lutz et al., 2013), which has been interpreted as either an empathic resonance with the painting’s content or imagery and mimicry of artistic gestures. Referring back to early psychological theories, Helmholtz (1868/1995) combined the ideas that artists test and explore the visual system with a theory of vision. Artists, he claimed, are particularly good at observing their sensuous impressions (data of sensation) and at figuring out which patterns trigger which interpretations. Although many visual scientists have given up Helmholtz’s theory of vision, the idea that artists test and explore the visual system has not really been abandoned (Pöppel et al., 2013). On the contrary, it allows for a more detailed and discriminating version of the same idea.

At the neural level, a considerable number of brain imaging studies on aesthetic experience demonstrate the involvement of brain regions, like the ventral striatum and medial prefrontal cortex, which are activated by reward and positive emotion (Cinzia and Vittorio, 2009; Chatterjee, 2011; Nadal and Pearce, 2011; Pearce et al., 2016). The default-mode network (DMN) is also involved when processing paintings of high aesthetic preference (Vessel et al., 2012) or of higher predictability in inferring meaningful content (Silveira et al., 2012) and has been proposed as a delayed aesthetic network (Cela-Conde et al., 2013). The DMN displays the highest activation during the resting state and task-dependent, lower activation levels (Raichle and Snyder, 2007). Overlapping with the cortical midline structures, parts of the DMN may be neural correlates for self-relevant processes. Particularly posterior parts, i.e., the posterior cingulate cortex (PCC) and adjacent precuneus, supposedly correspond to mental processes while integrating external information into a self-referential context (Northoff and Bermpohl, 2004). These processes seem to be culturally sensitive (Han and Northoff, 2008). According to recent enactive accounts of aesthetic experience (Xenakis and Arnellos, 2014, 2015), aesthetic experience arises as a result of the interaction between the viewer and the object. Aesthetic experience is also an embodied phenomenon directly linked to adaptation and aesthetic perception and helps cope with the environment. Aesthetic experience arises when the viewer interacts with both uncertain physical and cultural contexts. A more inclusive understanding of aesthetic experiences in diverse cultures has to be developed. Cultural aesthetics requires an empirical inquiry into the kinds and varieties of experiences associated with artistic activities as they are understood in different cultures.

Regarding cultural aspects, Western and Eastern artists tend to use different perspectives to represent the visual world. Western landscape paintings have been rather precise reproductions of visual surroundings since the Renaissance (Kubovy, 1986), while Eastern landscape paintings, such as Chinese paintings, have a dynamic quality that integrates successive time windows (Bao et al., 2015) and are expected to convey the experience of “being in nature” rather than “seeing nature.” This has led to an arrangement of spatial information in a vertical manner with multi-layers on top of each other in a scroll form (Sullivan, 1984; Cameron, 1993; Law, 2011; Tyler and Chen, 2011). Considering cultural and historical contexts in which painting styles evolved, Western artists applied more logic and mathematics (Kline, 1964), like geometric perspective. Prevailing Buddhist and Taoist influences among Chinese artists could adequately explain the emergence of landscape painting in the Southern Song period and its persistence throughout the Ming dynasty (Golas, 2014). Besides the different artistic approaches employed by Western and Eastern painters, viewers from these two cultural groups also have distinctly different aesthetic reactions to the same artistic visual representations. Both Western and Chinese participants gave higher aesthetic ratings to paintings from their own compared to the other culture (Bao et al., 2016). One explanation might be that repeated exposure to artworks (Zajonc, 2001) promotes the development of concepts of beauty, but also of culturally transmitted values and beliefs. A given cultural value system is internalized by members of the culture, and those who internalize that system display habitual ways of thinking and behaving. Certain artworks can trigger a culturally specific feeling of identity (Pöppel, 2010). We suggest that the cultural environment in which the individual is socialized can account for the production and appreciation of art.

The current study aimed at further investigating aesthetic processes in the context of culture. We used cross-cultural neuroimaging to measure neural activity in native Chinese and Europeans living in Germany while they viewed Western and traditional Chinese landscape paintings. Based on behavioral findings of cross-cultural differences, we predicted that distinct brain-activation patterns can be observed which correspond to aesthetic preference of one’s own vs. the other culture.

## Materials and Methods

### Participants

Thirty-one volunteers, including 16 Europeans (7 females; mean age = 24.45 years, *SD* = 4.51 years) and 15 Chinese (8 females; mean age = 27.38 years, *SD* = 1.78 years) from the Ludwig-Maximilian University in Munich (LMU), took part in the experiment after giving informed written consent. The Chinese students are from mianland China. None of them had studied in Germany for more than 4 years when they participated in this study. All participants had normal or corrected-to-normal visual acuity and color vision, and had no history of neurological disease. To minimize the confounding effect of art education, we defined previous formal art training or art-expertise as an exclusion criteria. None of these volunteers was an art expert. Participants were asked about their preferred painting style before the experiment. They generally did not show any specific interest in a certain painting style. The study was approved by the Ethics Committee of the LMU in agreement with the Declaration of Helsinki.

### Materials

**FIGURE 1:**
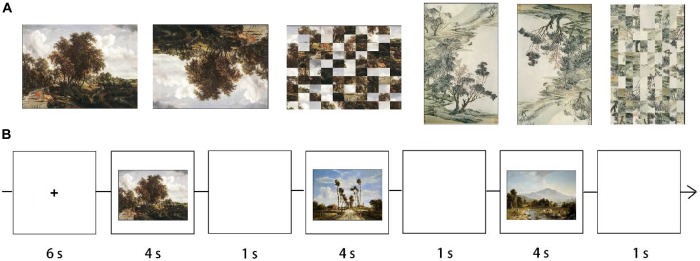
**(A)** Examples of the six types of stimuli used in the experiment. From left, original Western landscape painting, upside-down Western landscape painting, scrambled Western landscape painting, original Eastern landscape painting, upside-down Eastern landscape painting, scrambled Eastern landscape painting. **(B)** A Sample block in original Western painting condition. The image stimulus duration was 4 s, with an interstimulus interval (ISI) of 1 s. A fixation cross was shown during the intervals between the blocks when no image was displayed.

Twenty-one Western oil landscape paintings and 21 traditional Chinese landscape paintings were selected from the stimuli set used by Bao et al. (2016). The stimuli set was assembled by the authors in consultation with an art specialist and was completed by using http://www.artcyclopedia.com as a search engine and was then directed to Wikimedia Commons. The contents of the landscapes mainly included the sky, mountains, rivers, trees, flowers, meadows, houses, and boats. The paintings were chosen from a variety of historical periods (from the 9th to the 18th centuries). We believe to have chosen a representative sample of paintings from the two cultural environments. All paintings were prepared in uncompressed bitmap file format, and the image dimensions varied. These paintings are referred to as Western or Eastern originals. For visual baseline, a scrambled version for each original landscape painting was created in MATLAB by dividing the original paintings into10^∗^10 pixel units and then randomly shuffling the units to produce the scrambled stimuli. The paintings that were created using this process are referred to as Western and Eastern scrambled control condition, as they retained the overall colors of the original paintings while lacking perceptual/visual recognition. Each original painting was inverted to create an upside-down version of Western and Eastern landscape paintings. The inversion was meant to disrupt the content-related perception of the paintings. Six types of paintings were created, resulting in 126 paintings in all (see Figure 1A).

### Procedure

All stimuli were projected onto a translucent screen through a video projector that participants could view from inside the scanner via a head-coil-compatible mirror system. A classic block design was utilized as the experimental procedure. The six types of paintings constituted six experimental conditions, each including 21 paintings separated into seven blocks. Each block consisted of three paintings of the same type; there were 42 blocks altogether. The order of all blocks was pseudo-randomized. Each block started with a fixation cross lasting for 6 s, followed by the successive presentation of three paintings. Each painting was framed on a black background and presented for 4 s followed by a 1 s interval of black screen (see Figure 1B). In a previous behavioral study conducted by our research group which asked participants to give ratings on beauty of Western and Chinese paintings (Bao et al., 2016), 4 s was long enough for participants to give reliable responses. Participants were instructed to view the paintings in a subjective and engaged manner, without giving any explicit response. The instruction was, “Please pay attention to the paintings shown on the screen, experience the mood of the work and the feelings it evokes, and focus on its colors, tones, composition, and shapes.” To ensure sufficient attention to these paintings, participants were informed they had to fill out a questionnaire after the functional-imaging session. The decision not to collect behavioral responses in the scanner was made to facilitate maximal concentration on viewing the paintings. After the completion of the scans, each subject viewed the original paintings presented in the same block structure as in the scanner; stimulus presentation was self-paced. Participants were required to give ratings on a Likert-type Scale ranging from 1 to 7. The questions were as follows:

1. I find this painting calming (1).
  I find this painting exciting (7).
2. This painting makes me feel negative (1).
  This painting makes me feel positive (7).
3. I do not like this painting at all (1).
  I like this painting very much (7).
4. This painting is very ugly (1).
  This painting is very beautiful (7).
5. In the hospital, I would not like to hang this painting on the wall (1).
  In the hospital, I would like it a lot to hang this painting on the wall (7).
6. I haven’t seen this painting before, and it feels unfamiliar (1).
  I have seen this painting before, and it feels familiar to me (7).
7. I don’t have any feelings about this painting (1).
  I have strong feelings about this painting (7).
8. I feel like I have a distance to this painting (Outsider perspective) (1).
  I feel like I belong in this painting. (Insider perspective) (7).

The above eight questions are associated with the following mental processes, respectively: arousal, valence, preference, beauty, relaxation, familiarity, empathy, and object-related absorption. All instructions, stimulus materials, and questionnaires were given in German and then back-translated from German into Chinese by a bilingual speaker (Brislin, 1970).

### Image Acquisition

Brain imaging data was obtained with a 3T MRI scanner with a standard head coil at the university hospital of the LMU in Munich. For BOLD signals, T2^∗^-weighted EPI sequences were used [repetition time (TR) = 2500 ms; echo time (TE) = 30 ms; flip angle = 90°; acquisition matrix = 80 × 80; slice thickness = 3 mm, no gap between slices]. In total, one run of 358 functional volumes was acquired for each subject. Structural data was acquired with a T1-weighted scan of each participant’s brain anatomy (1 mm × 1 mm × 1 mm; 240 × 240 matrix, field-of view = 220 mm).

### Data Analysis

The behavioral data analysis was performed using SPSS for Windows (version 21.0). Paired *t*-tests were calculated for each question to compare the ratings on Western and Eastern paintings in European and Chinese groups separately. All neuroimaging data were preprocessed and analyzed using SPM12 (Statistical Parametric Mapping V12^1^). For each participant, the first eight volumes were removed to allow for T1-equilibration effects. The remaining 350 functional scan volumes were subjected to spatial realignment to correct for head motion. In further preprocessing analysis, the mean functional image was co-registered to the anatomical image, normalized to the Montreal Neurological Institute (MNI) template provided in SPM12, and spatially smoothed with an 8 mm Gaussian kernel.

The task was modeled as a block design. Using a two-level procedure, we conducted a random-effects fMRI data analysis. First, individual events were modeled by a hemodynamic response function. By using the general linear model, we obtained parameter estimates for each condition and each subject and then acquired statistical parametric maps of the *t*-statistic resulting from linear contrasts of the original and up-side-down conditions compared with their corresponding control conditions (original Western – scrambled Western, original Eastern – scrambled Eastern, upside-down Western – Scrambled Western, upside-down Eastern – Scrambled Eastern). Next, for the group analysis these individual contrast images were entered in a second-level analysis treating subjects as a random effect. The average BOLD response across the brain while viewing Western paintings was compared to Eastern paintings in both original and up-side-down conditions with paired *t*-tests. Reversed comparisons (Eastern paintings – Western paintings) were also conducted. For these *t*-tests, significant voxels initially passed a voxel-wise statistical threshold of *p* ≤ 0.01, and a cluster-level threshold was obtained at the family-wise-error (FWE)-corrected statistical significance level of *p* < 0.05.

## Results

### Behavioral Results

For the European group, ratings for each question are shown in Table 1. Paired *t*-tests revealed that there was no significant difference between original Western and Eastern landscape paintings in average familiarity: *t*(15) = 1.07, *p* = 0.301, *ES* = 0.214. However, compared with Eastern paintings, Western paintings were rated significantly higher on valence [*t*(15) = 4.01, *p* = 0.001, *ES* = 1.276], preference [*t*(15) = 3.75, *p* = 0.002, *ES* = 1.036], beauty [*t*(15) = 3.62, *p* = 0.003, *ES* = 0.938], relaxation [*t*(15) = 4.18, *p* = 0.001, *ES* = 0.842], empathy [*t*(15) = 4.84, *p* = 0.001, *ES* = 1.093], and object-related absorption [*t*(15) = 4.01, *p* = 0.002, *ES* = 0.968], but lower on arousal [*t*(15) = 2.63, *p* = 0.019, *ES* = 0.703].

**Table 1:** Means and standard deviations of ratings on each question for Western and Eastern landscape paintings by European participants.

	Arousal	Valence	Preference	Beauty	Relaxation	Familiarity	Empathy	Object-related absorption
Western	3.20	5.00	4.95	5.00	4.01	2.20	4.35	3.86
	(0.21)	(0.14)	(0.15)	(0.14)	(0.28)	(0.27)	(0.20)	(0.28)
Eastern	3.72	4.17	4.19	4.32	3.05	1.94	3.32	2.79
	(0.16)	(0.18)	(0.21)	(0.22)	(0.30)	(0.33)	(0.26)	(0.27)

Ratings from the Chinese group are shown in Table 2. Paired *t*-tests indicated that preference levels and beauty levels were not significantly different between traditional Chinese and Western landscape paintings [*t*(14) = 0.55, *p* = 0.58, *ES* = 0.037; *t*(14) = 1.36, *p* = 0.18, *ES* = 0.097]. However, traditional Chinese landscape paintings had significantly higher ratings in relaxation [*t*(14) = 2.50, *p* = 0.01, *ES* = 0.169] and familiarity [*t*(14) = 2.48, *p* = 0.014, *ES* = 0.143], but lower ratings in arousal [*t*(14) = 3.54, *p* < 0.001, *ES* = 0.226] and valence [*t*(14) = 5.58, *p* = 0.001, *ES* = 0.407] compared with Western landscape paintings. There was also a marginally significant effect on empathy [*t*(14) = 1.89, *p* = 0.059, *ES* = 0.145], but object-related absorption did not reach a significant level [*t*(14) = 0.84, *p* = 0.403, *ES* = 0.061].

**Table 2:** Means and standard deviations of ratings on each question for Western and Eastern landscape paintings by Chinese participants.

	Arousal	Valence	Preference	Beauty	Relaxation	Familiarity	Empathy	Object-related absorption
Western	3.73	4.39	4.47	4.61	4.03	2.73	3.80	3.68
	(0.10)	(0.08)	(0.07)	(0.07)	(0.09)	(0.10)	(0.08)	(0.09)
Eastern	3.32	3.73	4.52	4.73	4.31	2.98	4.00	3.78
	(0.10)	(0.10)	(0.07)	(0.07)	(0.08)	(0.09)	(0.08)	(0.09)

We performed an additional two-way analysis of variance (ANOVA) with gender as a between-subject variable for each question in both cultural groups. There was no interaction of gender by question, nor a main effect of gender (all *p* > 0.1).

### fMRI Results

**FIGURE 2:**
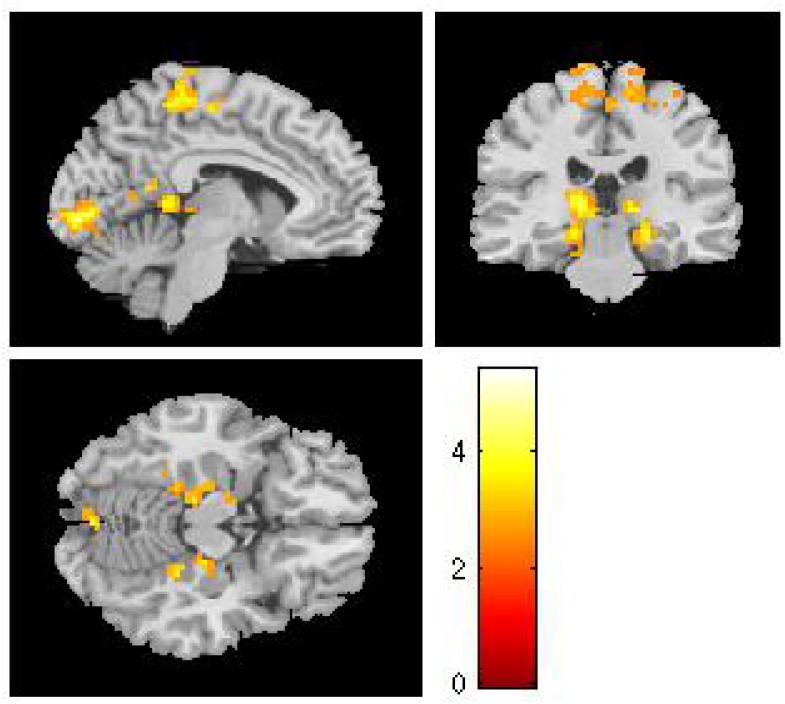
In the European group, comparisons of the original Western vs. original Eastern paintings revealed activation in the calcarine sulcus, the paracentral lobule, the posterior cingulate cortex, the hippocampus and the fusiform gyrus.

**FIGURE 3:**
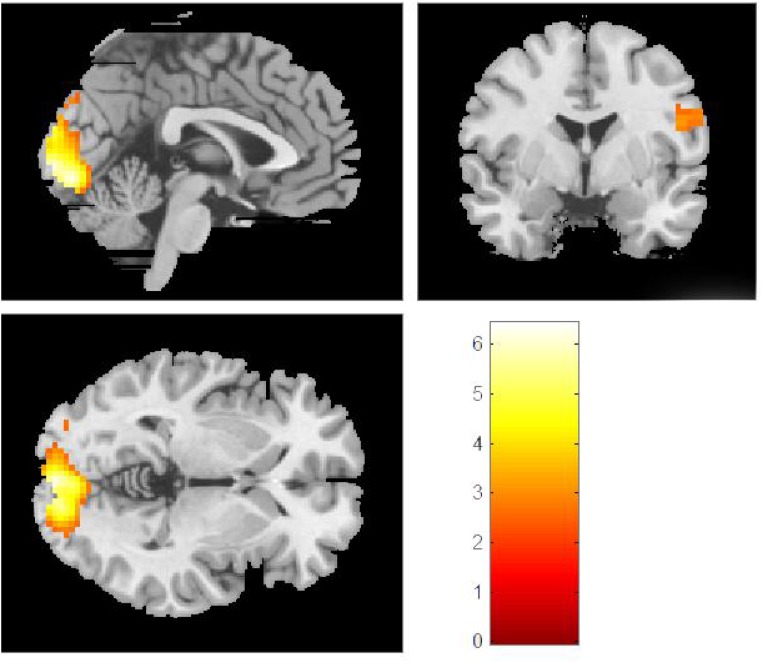
In the Chinese group, comparisons of the original Eastern vs. original Western paintings revealed activation in the right cuneus, the bilateral calcarine sulcus, the left lingual gyrus, the right postcentral gyrus, and the right superior parietal lobe.

For each cultural group, we created whole-brain activation maps by contrasting the group-level brain response to viewing Western landscape paintings with the responses to Eastern landscape paintings both in original and in upside-down versions. European and Chinese participants exhibited distinct neural response patterns. For the European group, the analysis of original Western paintings vs. original Eastern paintings revealed a network of regions distributed across the calcarine sulcus, i.e., the primary visual area, the supplementary motor area (SMA), the PCC, the hippocampus, and the fusiform gyrus (see Table 3 and Figure 2). The reverse comparison (original Western paintings vs. original Eastern paintings) revealed no significant activation. For the Chinese group, greater neural activity was observed in the right cuneus, the bilateral calcarine cortex, the left lingual gyrus, the right postcentral gyrus, and the right superior parietal lobule in response to Eastern paintings compared to Western paintings (Table 4 and Figure 3). No significant activation was found in the reverse comparison (original Western paintings vs. original Eastern paintings). When comparing the upside-down Western paintings vs. upside-down Eastern paintings or upside-down Eastern paintings vs. upside-down Western paintings, there were no significant differences detected in either cultural group.

**Table 3:** Location of brain regions that respond to comparison of original Western paintings vs. original Eastern paintings by European participants.

Brain regions	MNI coordinates			Z scores	Number of voxels
	*x*	*y*	*z*		
**Western – Eastern original paintings**					
L Calcarine Sulcus	−9	−88	−1	4.42	246
R Calcarine Sulcus	12	−85	2	3.98	
R Paracentral Lobule	9	−28	62	3.89	442
L Paracentral Lobule	−15	−22	80	3.77	
Posterior Cingulate	0	−52	11	3.67	616
L Hippocampus	−18	−28	−10	3.67	
L Fusiform Gyrus	−30	−43	−19	3.65	

*The reverse comparison (original Eastern vs. Western paintings) did not reach significance. Regions are designated using the MNI coordinates. L indicates the left hemisphere.*

**Table 4:** Location of brain regions that respond to comparison of original Eastern paintings vs. original Western paintings by Chinese participants.

Brain regions	MNI coordinates			Z scores	Number of voxels
	*x*	*y*	*z*		
**Eastern – Western original paintings**					
L Lingual Gyrus	−3	−85	−7	4.99	1168
R Calcarine Sulcus	−3	−94	−1	4.82	
R Cuneus	9	−94	17	4.75	
R Postcentral gyrus	33	−31	47	3.53	187
R Superior parietal lobule	33	−49	59	3.40	

*The reverse comparison (original Western vs. Eastern paintings) did not reach significance. Regions are designated using the MNI coordinates. L indicates the left hemisphere.*

To exclude the possible confounding factor of gender, a single regressor representing the gender was added to the design matrix used in the fMRI data analysis. This revealed no statistical significance (*p* > 0.1); our data do not support that gender is correlated with BOLD responses. The differences observed in brain activations are not explained by gender.

## Discussion

We investigated the neural correlates of viewing Western and Eastern landscape paintings in Western and Eastern participants with fMRI. We demonstrated a cultural difference in neural responses underlying aesthetic appreciation; participants showed stronger brain activation to artistic expressions from their own cultural systems. On the behavioral level, participants from the different cultural groups expressed distinct aesthetic judgments from the two different cultures. The behavioral results, however, did not support our expectation that participants would show an overall more positive aesthetic appreciation for landscape paintings from their own culture. European participants rated Western landscape paintings when compared to Eastern landscape paintings higher on valence, preference, beauty, relaxation, empathy, object-related absorption, and lower on arousal. Chinese participants did not express a general preference for Eastern landscape paintings; they gave compared to Western paintings only higher ratings in the domain of relaxation. Despite the absence of a behavioral evidence of reversed aesthetic preference patterns (which does not prove the evidence of absence), we observed greater brain activation in participants for landscape paintings from their own culture. The absence of an effect when comparing the upside-down versions of the paintings highlights the content dependency of the viewed paintings. One can conclude that the observed differences are not based on bottom-up processing of the physical properties of the paintings, but that pictorial properties of the paintings extracted on a level of higher cognitive processing are responsible.

When the European participants viewed original Western as compared to Eastern landscape paintings, the analysis revealed higher BOLD activation levels in the primary visual cortex, the SMA, the PCC, and the hippocampus. The SMA, which has motor and sensory functions (Jeannerod, 2001), counts as one of the commonly activated brain structures when viewing artworks (Vartanian and Skov, 2014; Boccia et al., 2016). Our results correspond to previous evidence relating exposure to aesthetic vs. non-aesthetic visual stimuli to sensory-motor processes (Freedberg and Gallese, 2007; Lutz et al., 2013) and may well indicate empathic resonance with paintings from one’s own cultural background. An enactive understanding of perception (Xenakis and Arnellos, 2014, 2015) suggests aesthetic behavior as emerging from the structural coupling of the viewer with his/her environment. The higher neural activation when viewing Western paintings is related to the viewer’s embodiment, as posited by enactivists, and to the whole spectrum of abilities available in human socio-cultural practices.

The most likely explanation for why Western paintings elicited higher activation in the fusiform gyrus in the European group is that this structure is known to mediate object recognition. When observers view a work of art, they do not see, for instance, a canvas or a statue on an abstract level; rather, they react to what it represents (e.g., a seascape, a tower). Compared to traditional Chinese landscape paintings, Western landscape paintings have clear-cut shapes similar to hard-edge paintings. In Chinese paintings, an object’s edge is ill-defined and disappears or fades into another object or into the background (Cahill, 1960; Zong, 2007). According to Wölfflin (1950), soft-edge paintings involve blurred boundaries and ill-defined forms, whereas hard-edge paintings involve well-defined forms and rich narrative details. Since the left fusiform gyrus probably plays a role in shape processing (Starrfelt and Gerlach, 2007), viewing Western landscape paintings invoked more fine-grained shape analysis than Chinese paintings, thus causing greater activation in the fusiform gyrus. This explanation is consistent with previous observations that activation in the fusiform gyrus can be attributed to object recognition in realistic paintings, including faces (Grill-Spector and Malach, 2004) and pictorial scenes (Siebörger et al., 2007).

The right cuneus, the bilateral calcarine cortex, and the left lingual gyrus were activated in the comparison of aesthetic appreciation of Eastern paintings vs. Western paintings by Chinese participants. Activation of the lingual gyrus has been linked to the encoding of complex images. Chatterjee et al. (2009) argued that activation of the lingual gyrus represents its sensitivity to “beauty,” which corresponds with the present findings. Mizokami et al. (2014) showed that the left lingual gyrus and the bilateral cuneus may be associated with aesthetic judgments of representational paintings, which also supports our findings. Previous studies have related the cuneus to the aesthetic appreciation of beauty (Cupchik et al., 2009; Mizokami et al., 2014). The stronger activation in these brain structures may be explained by higher aesthetic sensitivity to stimuli with high visualization demands. Of particular interest is activation specific to the Chinese paintings localized within the calcarine sulcus corresponding to the primary visual cortex. More specifically, activation was localized to the mid- to anterior parts of the calcarine sulcus, corresponding to the mid-level and peripheral visual field representations (Sereno et al., 1995).

The higher activation levels in the primary visual cortex in both cultural groups not only indicate stronger bottom-up visual processing of paintings from one’s own culture. The simultaneous involvement of the PCC in the European group, which has cortical connections to visual areas in feedforward, as well as feedback projections (Keil et al., 2009), also suggests stronger top-down processing, i.e., higher-level cortical areas modulate neural activity on the sensory-input levels. For the Chinese group, stronger activation was localized in the superior parietal lobule, a neural region known to support the manipulation of information in working memory (Koenigs et al., 2009). Top-down processes originating in superior parietal areas may contribute to the aesthetic perception of paintings and their maintenance in the “mind’s eye” (Mechelli et al., 2004).

Previous studies suggested that aesthetic experience is a function of the interaction between top-down and bottom-up processing (Leder et al., 2004; Cupchik et al., 2009; Redies, 2015). However, it is difficult to delineate the exact projecting pathway of feed-forward and feedback signals with the current experimental settings. Support for a stronger involvement of top–down information processing for paintings from one’s own cultural background is the additional engagement of the hippocampus, a region that has consistently been related to episodic memory retrieval (Eichenbaum et al., 1996, 2007; Brown and Aggleton, 2001). Paintings from one’s own culture might be integrated into a frame of prior experiences or expectations to a higher degree due to their fit with culturally imprinted cognitive schemata, thereby linking aesthetic appreciation to one’s own previous experiences. This could determine the relationship between cognition and action, thereby generating a value-oriented experience of high complexity (e.g., aesthetic appreciation), which motivates human social behavior to be related to certain types of socio-cultural expressions (e.g., appreciating or even creating/producing artworks).

We observed activation in the PCC in European participants when comparing Western to Eastern landscape paintings. This region has emerged as a key component of the DMN (Mason et al., 2007; Christoff et al., 2009; Andrews-Hanna et al., 2010), a highly interconnected network of brain regions that has been associated with self-referential mental processing (Northoff et al., 2006). More specifically, the PCC is active during tasks that involve integrating external stimuli in a self-referential manner (Northoff and Bermpohl, 2004), as well as jn autobiographical memory (Buckner and Carroll, 2007). The PCC responds to self-relevant information even when there is no explicit requirement to evaluate self-relevance (Moran et al., 2009; Reniers et al., 2012). In aesthetic experiences, individual taste in art can be considered as highly subjective, connected with personal pleasure, and can be considered a part of the personal self (Zaytseva et al., 2014). Previous research also provides evidence of higher activation levels within the DMN associated with intense aesthetic experience and interpreted as personal relevance of these stimuli (Vessel et al., 2012). When analyzing temporal dynamics of aesthetic appreciation, it has been suggested that the DMN involvement is a core part of general aesthetic experience, as represented in a delayed aesthetic network (Cela-Conde et al., 2013). The current study demonstrated that an inclusion of a cultural context was a modulator of these processes. Our results support previous findings of culturally sensitive information processing within the DMN (Han and Northoff, 2008). In Chinese group, we found evidence for increased activation in the right postcentral gyrus. This is noteworthy given that previous studies revealed the right postcentral gyrus to be activated in response to self-vs. other-perspective taking in social cognition (Ruby and Decety, 2004; Adams et al., 2010). Because the postcentral gyrus mediates somatosensory experience, the results might be explained by motor resonance created in the viewer when viewing paintings from his/her own culture. This activation, however, was not in a common area found in the previous tests of aesthetic appreciation and requires further empirical corroboration before firm conclusions can be drawn.

Why didn’t Chinese participants show any activation related to self-referential information processing? There are two possible explanations. Our experimental paradigm was comparatively subtle. Pronounced cultural differences in the neural processing may emerge under more demanding conditions, verifying the expected bias of the Eastern cultural group to show preferential processing of artworks in self-related brain regions similar to the Western groups. An alternate explanation is that Asian societies are changing rapidly, and that the young Chinese participants have internalized Western values up to the point that they no longer display behavioral patterns which are characteristic for Asian cultures. Even if this is the case, the neuroimaging results clearly demonstrate different patterns for Eastern and Western participants, with a bias toward higher brain activation for one’s own cultural artworks, a finding supporting the cultural/aesthetic framework proposed by Bao et al. (2016).

Our findings provide neuroimaging evidence for cultural biases in the processing of visual artworks. Visual aesthetic studies make us aware that “seeing” has a history; sensory experiences are socially, culturally, and historically embedded. It is not possible to speak of pure perception as sensation untouched by past experiences, education, and cultural background. Since our self-identity is influenced by the cultural background we inhabit, the “belonging” moments should occur with greater frequency when artists of the presented paintings and viewers who appreciate the paintings share the same cultural background. Members of different cultural groups are repeatedly exposed to various examples of visual images from their respective cultures, and they may implicitly gain knowledge (Pöppel and Bao, 2011) about the dominant aesthetic representation of the world. As mental representations of the world influence the perception of visual information, a cultural framing effect is probably implemented on the neural level and determines the implicit information processing of visual stimuli in particular and sensory stimuli in general. In this way, different cultural groups produce a “sensory model” of the meanings and values associated with their sensory perceptions. We propose that culturally distinct behaviors and thoughts can be construed as differences in aesthetic appreciation that affect neural functions. The authenticity of aesthetic experience, through its directness and immediacy, provides a powerful means of reappraising cultural experience by digging beneath the layers of accrued meanings and cognitive habits.

## Conclusion

In conclusion, our results highlight culture as a modulating factor in the visual perception of aesthetic stimuli. Aesthetic experiences are aligned with the human cognitive architecture which is based on the principles of efficiency and prediction. This indicates a cultural framing effect, which corresponds to the mind, as well as to brain states, and determines implicit information processing. Our findings support the notion that visual experiences are biased by cultural factors. We engage aesthetically with artwork in its different expressions (e.g., visual art, poetry, music) and also with different physical environments (e.g., untouched nature, architecture, or different kinds of urban surroundings). Environments define specific perceptual features as characteristic for a particular human culture at a given time resulting in typical aesthetic appreciation. Beauty really is in the “eye” of the beholder.

## Ethics Statement

This study was approved by the ethical committee of Ludwig-Maximilian University Munich, in agreement with the Declaration of Helsinki.

## Author Contributions

YB and EP conceived and designed the study. TY, SS, and AF acquired the data. TY and SS analyzed and discussed the data. TY and SS drafted the manuscript. MP, TS, YB, and EP critically reviewed the manuscript.

## Conflict of Interest Statement

The authors declare that the research was conducted in the absence of any commercial or financial relationships that could be construed as a potential conflict of interest.

## Footnotes

1 http://www.fil.ion.ucl.ac.uk/spm

## References

[B1] Adams R. B. Jr. Rule N. O. Franklin R. G. Jr. Wang E. Stevenson M. T. Yoshikawa S. (2010). Cross-cultural reading the mind in the eyes: an fMRI investigation. *J. Cogn. Neurosci.* 22 97–108. 10.1162/jocn.2009.21187 19199419

[B2] Andrews-Hanna J. R. Reidler J. S. Sepulcre J. Poulin R. Buckner R. L. (2010). Functional-anatomic fractionation of the brain’s default network. *Neuron* 65 550–562. 10.1016/j.neuron.2010.02.005 20188659 PMC2848443

[B3] Bao Y. Pöppel E. Wang L. Lin X. Yang T. Avram M. (2015). Synchronization as a biological, psychological and social mechanism to create common time: a theoretical frame and a single case study. *Psych J.* 4 243–254. 10.1002/pchj.119 26663630

[B4] Bao Y. von Stosch A. Park M. Pöppel E. (2017a). Complementarity as generative principle: a thought pattern for aesthetic appreciations and cognitive appraisals in general. *Front. Psychol.* 8:727. 10.3389/fp-syg.2017.00727 28536548 PMC5422519

[B5] Bao Y. Yang Y. Zhang J. Zhang J. Lin X. Paolini M. (2017b). The “Third Abstraction” of the Chinese artist LaoZhu: neural and behavioral indicators of aesthetic appreciation. *Psych J.* 6 110–119. 10.1002/pchj.167 28660742

[B6] Bao Y. Yang T. Lin X. Fang Y. Wang Y. Pöppel E. (2016). Aesthetic preferences for Eastern and Western traditional visual art: identity matters. *Front. Psychol.* 7:1596. 10.3389/fpsyg.2016.01596 27812339 PMC5071313

[B7] Boccia M. Barbetti S. Piccardi L. Guariglia C. Ferlazzo F. Giannini A. M. (2016). Where does brain neural activation in aesthetic responses to visual art occur? Meta-analytic evidence from neuroimaging studies. *Neurosci. Biobehav. Rev.* 60 65–71. 10.1016/j.neubiorev.2015.09.009 26619805

[B8] Brislin R. W. (1970). Back-translation for cross-cultural research. *J. Cross Cult. Psychol.* 1 185–216. 10.1177/135910457000100301

[B9] Brown M. W. Aggleton J. P. (2001). Recognition memory: what are the roles of the perirhinal cortex and hippocampus? *Nature Rev. Neurosci.* 2 51–61. 11253359 10.1038/35049064

[B10] Buckner R. L. Carroll D. C. (2007). Self-projection and the brain. *Trends Cogn. Sci.* 11 49–57. 10.1016/j.tics.2006.11.004 17188554

[B11] Cahill J. (1960). *Chinese Painting.* Geneva: Skira.

[B12] Cameron A. S. (1993). *Chinese Painting Techniques.* Mineola, NY: Dover Publications.

[B13] Cela-Conde C. J. García-Prieto J. Ramasco J. J. Mirasso C. R. Bajo R. Munar E. (2013). Dynamics of brain networks in the aesthetic appreciation. *Proc. Natl. Acad. Sci. U.S.A.* 110(Suppl. 2), 10454–10461. 10.1073/pnas.1302855110 23754437 PMC3690613

[B14] Chatterjee A. (2011). Neuroaesthetics: a coming of age story. *J. Cogn. Neurosci.* 23 53–62. 10.1162/jocn.2010.21457 20175677

[B15] Chatterjee A. Thomas A. Smith S. E. Aguirre G. K. (2009). The neural response to facial attractiveness. *Neuropsychology* 23 135–143. 10.1037/a0014430 19254086

[B16] Chatterjee A. Vartanian O. (2014). Neuroaesthetics. *Trends Cogn. Sci.* 18 370–375. 10.1016/j.tics.2014.03.003 24768244

[B17] Christoff K. Gordon A. M. Smallwood J. Smith R. Schooler J. W. (2009). Experience sampling during fMRI reveals default network and executive system contributions to mind wandering. *Proc. Natl. Acad. Sci. U.S.A.* 106 8719–8724. 10.1073/pnas.0900234106 19433790 PMC2689035

[B18] Cinzia D. D. Vittorio G. (2009). Neuroaesthetics: a review. *Curr. Opin. Neurobiol.* 19 682–687. 10.1016/j.conb.2009.09.001 19828312

[B19] Clark A. (2013). Whatever next? Predictive brains, situated agents, and the future of cognitive science. *Behav. Brain Sci.* 36 181–204. 10.1017/S0140525X12000477 23663408

[B20] Cupchik G. C. Vartanian O. Crawley A. Mikulis D. J. (2009). Viewing artworks: contributions of cognitive control and perceptual facilitation to aesthetic experience. *Brain Cogn.* 70 84–91. 10.1016/j.bandc.2009.01.003 19223099

[B21] Eichenbaum H. Schoenbaum G. Young B. Bunsey M. (1996). Functional organization of the hippocampal memory system. *Proc. Natl. Acad. Sci. U.S.A.* 93 13500–13507. 10.1073/pnas.93.24.13500 8942963 PMC33637

[B22] Eichenbaum H. Yonelinas A. P. Ranganath C. (2007). The medial temporal lobe and recognition memory. *Annu. Rev. Neurosci.* 30 123–152. 10.1146/annurev.neuro.30.051606.094328 17417939 PMC2064941

[B23] Freedberg D. Gallese V. (2007). Motion, emotion and empathy in esthetic experience. *Trends Cogn. Sci.* 11 197–203. 10.1016/j.tics.2007.02.003 17347026

[B24] Golas P. J. (2014). *Picturing Technology in China: From Earliest Times to the Nineteenth Century.* Hong Kong: Hong Kong University Press.

[B25] Graupmann V. Peres I. Michaely T. Meindl T. Frey D. Reiser M. (2013). Culture and its neurofunctional correlates when death is in mind. *Neurosci. Lett.* 548 239–243. 10.1016/j.neulet.2013.05.062 23752131

[B26] Grill-Spector K. Malach R. (2004). The human visual cortex. *Annu. Rev. Neurosci.* 27 649–677. 10.1146/annurev.neuro.27.070203.144220 15217346

[B27] Han S. Northoff G. (2008). Culture-sensitive neural substrates of human cognition: a transcultural neuroimaging approach. *Nat. Rev. Neurosci.* 9 646–654. 10.1038/nrn2456 18641669

[B28] Heidegger M. (1986). *Der Ursprung des Kunstwerkes.* Ditzingen: Reclam.

[B29] Helmholtz H. (1868/1995). “On the relation of optics to painting,” in *Science and Culture: Popular and Philosophical Essays*, ed. Cahan D. (Chicago, IL: University of Chicago Press), 279–308.

[B30] Jeannerod M. (2001). Neural simulation of action: a unifying mechanism for motor cognition. *Neuroimage* 14 S103–S109. 10.1006/nimg.2001.0832 11373140

[B31] Jucker J. L. Barrett J. L. (2011). Cognitive constraints on the visual arts: an empirical study of the role of perceived intentions in appreciation judgements. *J. Cogn. Cult.* 11 115–136. 10.1163/156853711X568716

[B32] Kawabata H. Zeki S. (2004). Neural correlates of beauty. *J. Neurophysiol.* 91 1699–1705. 10.1152/jn.00696.2003 15010496

[B33] Keil A. Sabatinelli D. Ding M. Lang P. J. Ihssen N. Heim S. (2009). Re-entrant projections modulate visual cortex in affective perception: evidence from Granger causality analysis. *Hum. Brain Mapp.* 30 532–540. 10.1002/hbm.20521 18095279 PMC3622724

[B34] Kline M. (1964). *Mathematics in Western Culture.* Oxford: Oxford University Press.

[B35] Koenigs M. Barbey A. K. Postle B. R. Grafman J. (2009). Superior parietal cortex is critical for the manipulation of information in working memory. *J. Neurosci.* 29 14980–14986. 10.1523/JNEUROSCI.3706-09.2009 19940193 PMC2799248

[B36] Kubovy M. (1986). *The Psychology of Perspective and Renaissance Art.* New York, NY: Cambridge University Press.

[B37] Latto R. (1995). *The Brain of the Beholder.* Oxford: Oxford University Press.

[B38] Law S. S. M. (2011). Being in traditional Chinese landscape painting. *J. Intercult. Stud.* 32 369–382. 25610386

[B39] Leder H. (2013). Next steps in neuroaesthetics: which processes and processing stages to study? *Psychol. Aesthet. Creat. Arts* 7:27 10.1037/a0031585

[B40] Leder H. Belke B. Oeberst A. Augustin D. (2004). A model of aesthetic appreciation and aesthetic judgments. *Br. J. Psychol.* 95 489–508. 10.1348/0007126042369811 15527534

[B41] Lutz A. Nassehi A. Bao Y. Pöppel E. Sztrókay A. Reiser M. (2013). Neurocognitive processing of body representations in artistic and photographic images. *Neuroimage* 66 288–292. 10.1016/j.neuroimage.2012.10.067 23123681

[B42] Mason M. F. Norton M. I. Van Horn J. D. Wegner D. M. Grafton S. T. Macrae C. N. (2007). Wandering minds: the default network and stimulus-independent thought. *Science* 315 393–395. 10.1126/science.1131295 17234951 PMC1821121

[B43] Mechelli A. Price C. J. Friston K. J. Ishai A. (2004). Where bottom-up meets top-down: neuronal interactions during perception and imagery. *Cereb. Cortex* 14 1256–1265. 10.1093/cercor/bhh087 15192010

[B44] Mizokami Y. Terao T. Hatano K. Hoaki N. Kohno K. Araki Y. (2014). Difference in brain activations during appreciating paintings and photographic analogs. *Front. Hum. Neurosci.* 8:478. 10.3389/fnhum.2014.00478 25071508 PMC4083828

[B45] Moran J. M. Heatherton T. F. Kelley W. M. (2009). Modulation of cortical midline structures by implicit and explicit self-relevance evaluation. *Soc. Neurosci.* 4 197–211. 10.1080/17470910802250519 19424905 PMC4532268

[B46] Nadal M. Pearce M. T. (2011). The Copenhagen Neuroaesthetics conference: prospects and pitfalls for an emerging field. *Brain Cogn.* 76 172–183. 10.1016/j.bandc.2011.01.009 21334125

[B47] Northoff G. Bermpohl F. (2004). Cortical midline structures and the self. *Trends Cogn. Sci.* 8 102–107. 10.1016/j.tics.2004.01.004 15301749

[B48] Northoff G. Heinzel A. De Greck M. Bermpohl F. Dobrony H. Panksepp J. (2006). Self-referential processing in our brain - a meta-analysis of imaging studies on the self. *Neuroimage* 31 440–457. 10.1016/j.neuroimage.2005.12.002 16466680

[B49] Palmer S. E. Schloss K. B. Sammartino J. (2013). Visual aesthetics and human preference. *Annu. Rev. Psychol.* 64 77–107. 10.1146/annurev-psych-120710-100504 23020642

[B50] Pearce M. T. Zaidel D. W. Vartanian O. Skov M. Leder H. Chatterjee A. (2016). Neuroaesthetics: the cognitive neuroscience of aesthetic experience. *Perspect. Psychol. Sci.* 11 265–279. 10.1177/1745691615621274 26993278

[B51] Pöppel E. (2010). “Perceptual identity and personal self: neurobiological reflections,” in *Personality From Biological, Cognitive, and Social Perspectives*, eds Maruszewski T. Fajkowska M. Eysenck M. M. (Clinton Corners, NY: Eliot Werner Publications), 75–82.

[B52] Pöppel E. Avram A. Bao Y. Graupmann V. Gutyrchik E. Lutz A. (2013). Sensory processing of art as a unique window into cognitive mechanisms: evidence from behavioral experiments and fMRI studies. *Procedia Soc. Behav. Sci.* 86 10–17. 10.1016/j.sbspro.2013.08.517

[B53] Pöppel E. Bao Y. (2011). “Three modes of knowledge as basis for intercultural cognition and communication: a theoretical perspective,” in *Culture and Neural Frames of Cognition and Communication*, eds Han S. P E.öppel (Heidelberg: Springer-Verlag), 215–231.

[B54] Raichle M. E. Snyder A. Z. (2007). A default mode of brain function: a brief history of an evolving idea. *Neuroimage* 37 1083–1090. 10.1016/j.neuroimage.2007.02.041 17719799

[B55] Redies C. (2015). Combining universal beauty and cultural context in a unifying model of visual aesthetic experience. *Front. Hum. Neurosci.* 9:218. 10.3389/fnhum.2015.00218 25972799 PMC4412058

[B56] Reniers R. L. Corcoran R. Völlm B. A. Mashru A. Howard R. Liddle P. F. (2012). Moral decision-making, ToM, empathy and the default mode network. *Biol. Psychol.* 90 202–210. 10.1016/j.biopsycho.2012.03.009 22459338

[B57] Ruby P. Decety J. (2004). How would you feel versus how do you think she would feel? A neuroimaging study of perspective-taking with social emotions. *J. Cogn. Neurosci.* 16 988–999. 10.1162/0898929041502661 15298786

[B58] Sereno M. I. Dale A. M. Reppas J. B. Kwong K. K. Belliveau J. W. Brady T. J. (1995). Borders of multiple visual areas in humans revealed by functional magnetic resonance imaging. *Science* 268 889–893. 10.1126/science.7754376 7754376

[B59] Siebörger F. T. Ferstl E. C. von Cramon D. Y. (2007). Making sense of nonsense: an fMRI study of task induced inference processes during discourse comprehension. *Brain Res.* 1166 77–91.3. 10.1016/j.brainres.2007.05.079 17655831

[B60] Silveira S. Bao Y. Wang L. Pöppel E. Avram M. Simmank F. (2015a). Does a bishop pray when he prays? And does his brain distinguish between different religions? *Psych J.* 4 199–207. 10.1002/pchj.116 26663626

[B61] Silveira S. Fehse K. Vedder A. Elvers K. Hennig-Fast K. (2015b). Is it the picture or is it the frame? An fMRI study on the neurobiology of framing effects. *Front. Hum. Neurosci.* 9:528. 10.3389/fnhum.2015.00528 26528161 PMC4602085

[B62] Silveira S. Gutyrchik E. Wetherell G. Bao Y. Pöppel E. Blautzik J. (2015c). Ceci n’est pas la mort: evidence for the recruitment of self-reference from surrealistic art under mortality salience. *Eur. J. Soc. Psychol.* 45 255–266. 10.1002/ejsp.2076 19097482

[B63] Silveira S. Graupmann V. Frey D. Blautzik J. Meindl T. Reiser M. (2012). Matching reality in the arts: self-referential neural processing of naturalistic compared to surrealistic images. *Perception* 41 569–576. 10.1068/p7191 23025160

[B64] Starrfelt R. Gerlach C. (2007). The visual what for area: words and pictures in the left fusiform gyrus. *Neuroimage* 35 334–342. 10.1016/j.neuroimage.2006.12.003 17239621

[B65] Sullivan M. (1984). *The Arts of China*, 5th Edn. Berkeley: University of California Press.

[B66] Tinio P. P. (2013). From artistic creation to aesthetic reception: the mirror model of art. *Psychol. Aesthet. Creat. Arts* 7:265 10.1037/a0030872

[B67] Tyler C. W. Chen C. C. (2011). Chinese perspective as a rational system: relationship to Panofsky’s symbolic form. *Chin. J. Psychol.* 53 371–391.

[B68] Vartanian O. Skov M. (2014). Neural correlates of viewing paintings: evidence from a quantitative meta-analysis of functional magnetic resonance imaging data. *Brain Cogn.* 87 52–56. 10.1016/j.bandc.2014.03.004 24704947

[B69] Vessel E. A. Starr G. G. Rubin N. (2012). The brain on art: intense aesthetic experience activates the default mode network. *Front. Hum. Neurosci.* 6:66. 10.3389/fnhum.2012.00066 22529785 PMC3330757

[B70] von Holst E. Mittelstaedt H. (1950). Das reafferenzprinzip. *Naturwissenschaften* 37 464–476. 10.1007/BF00622503

[B71] Wölfflin H. (1950). *Principles of Art History.* New York, NY: Dover Publications Inc.

[B72] Xenakis I. Arnellos A. (2014). Aesthetic perception and its minimal content: a naturalistic perspective. *Theor. Philos. Psychol.* 5:1038. 10.3389/fpsyg.2014.01038 25285084 PMC4168683

[B73] Xenakis I. Arnellos A. (2015). “Aesthetics as an emotional activity that facilitates sense-making: towards an enactive approach to aesthetic experience,” in *Aesthetics and the Embodied Mind: Beyond Art Theory and the Cartesian Mind-Body Dichotomy Contributions to Phenomenology*, ed. Scarinzi A. (Dordrecht: Springer), 245–259.

[B74] Zaidel D. W. (2015). Neuroesthetics is not just about art. *Front. Hum. Neurosci.* 9:80. 10.3389/fnhum.2015.00080 25741273 PMC4330786

[B75] Zajonc R. B. (2001). Mere exposure: a gateway to the subliminal. *Curr. Dir. Psychol. Sci.* 10 224–228. 10.1111/1467-8721.00154

[B76] Zaytseva Y. Gutyrchik E. Bao Y. Pöppel E. Han S. Northoff G. (2014). Self processing in the brain: a paradigmatic fMRI case study with a professional singer. *Brain Cogn.* 87 104–108. 10.1016/j.bandc.2014.03.012 24732954

[B77] Zeki S. (1999). *Inner Vision: An Exploration of Art and the Brain.* Oxford: University Press.

[B78] Zhao C. Zhang D. Bao Y. (2018). A time window of 3 seconds in the aesthetic appreciation of poems. *Psych J.* 7 51–52. 10.1002/pchj.194 29297978

[B79] Zhou B. Pöppel E. Wang L. Yang Y. Zaytseva Y. Bao Y. (2016). Seeing without knowing: operational principles along the early visual pathway. *Psych J.* 5 145–160. 10.1002/pchj.141 27678480

[B80] Zong B. (2007). *Shang Shui Hua Xu.* Jiangsu: Jiangsu Meishu.

